# Signs and symptoms in plantar vein thrombosis

**DOI:** 10.1590/1677-5449.202501092

**Published:** 2025-10-31

**Authors:** Steven Howard Yale, Halil Tekiner, Eileen Scott Yale

**Affiliations:** 1 University of Central Florida College of Medicine, Orlando, Florida, USA.; 2 Erciyes University School of Medicine, Kayseri, Türkiye.; 3 Nova Southeastern University Medical School, Kiran C. Patel College of Allopathic Medicine, Florida, USA.

To the Editor,

We wish to contribute further to the discussion initiated by França *et al*. in their paper, “Diagnosis of plantar vein thrombosis by vascular ultrasound: a case report,” by highlighting three historical clinical signs that may help raise suspicion for plantar vein thrombosis (PVT).^1^ As França *et al*. emphasize, PVT is rare and often underrecognized, with non-specific symptoms such as plantar pain, edema, and difficulty walking.^1^

Before the routine use of vascular ultrasound, early 20th-century clinicians described physical signs that, although not pathognomonic, could support consideration of lower extremity thrombosis when unexplained plantar pain is present.

Kurt Denecke (1903–1991) observed that after active foot flexion following prolonged bed rest, patients may develop sharp pain at the calcaneus within 24 hours. He regarded this as an early sign of thrombosis and advised assessing for pressure pain over the medial dorsum of the foot.^2^

Erwin Payr (1871–1946) described tenderness over the calcaneus on the medial plantar aspect and later over the posterior tibial vein. Passive dorsiflexion could induce pain, especially with simultaneous pressure on the deep calf veins. Payr hypothesized that thrombosis may begin at the *rete plantar venosum*. Within 24 hours, edema, calf induration, and resistance to passive dorsiflexion may appear.^3^

Robert Neumann (1902–1962) noted that thrombi often form where ligaments, tendons, or bone cross veins. He described “proximal” and “distal” plantar vein points resulting from segmentation in the plantar region, which may contribute to lateral PVT. These sites can cause vascular bundle entrapment.^4^

The rarity of PVT likely reflects historical underrecognition and modern preventive measures such as early mobilization. Nonetheless, these signs may help raise suspicion in patients with unexplained plantar pain after bed rest or immobilization and should prompt ultrasound confirmation.

## Data Availability

Data not reported or used: “Data sharing does not apply to this article, as no data were generated or analyzed.”
