# Supplementary material for: Signs and symptoms in plantar vein thrombosis
Source: J Vasc Bras. 2025 Oct 31;24:e20250109. doi: 10.1590/1677-5449.202501092 (PMC12642922; doi:10.1590/1677-5449.202501092)
Supplement: Related texts

# Reply: RESPONSE LETTER

**Authors:** Mariana Jordão França, Luciana Akemi Takahashi, Graciliano José França

**Affiliations:** 1 Universidade Positivo – UP, Curitiba, PR, Brasil.; 2 Universidade Federal do Paraná – UFPR, Curitiba, PR, Brasil.

Dear editor,

Initially, we would like to thank you for your pertinent comments on our manuscript, “Diagnosis of plantar vein thrombosis by vascular ultrasound: a case report,” published in the Jornal Vascular Brasileiro. We are grateful for the historical insights shared, which further enrich our understanding of plantar vein thrombosis.

We found the references to the classical clinical signs described by Denecke, Payr, and Neumann^1^ to be highly relevant. These physical findings may help raise early clinical suspicion for plantar vein thrombosis. As highlighted in your letter, the rarity of reported cases of plantar vein thrombosis may lead to underdiagnosis or misdiagnosis. In clinical scenarios involving unexplained plantar pain, edema, or difficulty walking, especially following a period of immobility or prolonged bed rest, awareness of these physical signs can assist physicians in considering plantar vein thrombosis in the differential diagnosis and in requesting a Doppler ultrasound, to assess vein compressibility.

We sincerely thank you again for your valuable contribution.

## References

[1] Denecke K. (1929). Der Plantarschmerz als Frühsymptom einer beginnenden Thrombose der unteren Extremität. München med Wchnschr.
